# DO PROPRIOCEPTIVE TRAINING STRATEGIES WITH DUAL-TASK EXERCISES POSITIVELY INFLUENCE GAIT PARAMETERS IN CHRONIC STROKE? A SYSTEMATIC REVIEW

**DOI:** 10.2340/jrm.v56.18396

**Published:** 2024-08-15

**Authors:** Michele VECCHIO, Rita CHIARAMONTE, Alessandro DE SIRE, Enrico BUCCHERI, Patrizia FINOCCHIARO, Dalila SCATURRO, Giulia Letizia MAURO, Matteo CIONI

**Affiliations:** 1 Department of Biomedical and Biotechnological Sciences, University of Catania, Catania; 2 Rehabilitation Unit, AOU Policlinico G. Rodolico-San Marco, Catania; 3 Physical and Rehabilitative Medicine, Department of Medical and Surgical Sciences, University of Catanzaro Magna Graecia, Catanzaro; 4 Research Center on Musculoskeletal Health, MusculoSkeletalHealth@UMG, University of Catanzaro Magna Graecia, Catanzaro; 5 Department of Surgery, Oncology and Stomatology, University of Palermo, Palermo; 6 Laboratory of Neuro-Biomechanics, Department of Biomedical and Biotechnological Sciences, University of Catania, Catania, Italy

**Keywords:** proprioception, stroke, gait analysis, rehabilitation, task performance, hemiplegia

## Abstract

**Objective:**

This study aims to assess the impact of proprioceptive training strategies with dual-task exercises on gait in people with chronic stroke.

**Study design:**

Systematic review.

**Patients:**

Chronic stroke.

**Methods:**

Searches were conducted in accordance with PRISMA guidelines and PICOS criteria. PubMed, Web of Science, and Scopus databases were systematically searched from November 2020 to February 2022, for eligible clinical trials. Two independent reviewers thoroughly screened potential articles for relevance and assessed the methodology quality. In accordance with the GRADE, PICOS criteria, and Cochrane risk of bias tools, the authors included articles concerning the effectiveness of dual-task in proprioceptive training on gait parameters in people with chronic stroke.

**Results:**

Of 3075 identified studies, 11 articles met the inclusion criteria: 7 were randomized clinical trials, 1 was not randomized, and 3 were observational studies. The overall quality of evidence, assessed using the GRADE framework, was high, indicating a high level of confidence in the systematic review’s findings. The papers involved 393 stroke patients; 241 underwent dual-task in proprioceptive training, with 152 participants in other stroke rehabilitation; within the dual-task group, 71 engaged in cognitive tasks, and 170 participated in motor tasks. dual-task in proprioceptive training improved gait speed, cadence, stride time, stride length, and step length. The best effects were observed with training 3 times a week for 4 weeks, with each session lasting 30 minutes, on speed, cadence, stride length, and step length.

**Conclusion:**

Current evidence suggests that proprioceptive training strategies with dual-task exercises improved walking abilities in people with chronic stroke. Specifically, it enhanced gait speed, a key indicator of clinical severity.

## LAY ABSTRACT

This study explored the unique training method that focuses on improving the sense of body positioning and movement, incorporating the concept of “doing two tasks” simultaneously. People recovering from a stroke often face challenges in walking and multitasking, such as while walking performing other tasks simultaneously. A problem with our sense of proprioception is common after a stroke, interfering with carrying out everyday activities like activities of daily living. Proprioception allows us to move more freely without consciously thinking about our environment. Our investigation involved reviewing 11 articles on this approach. The search results were promising; individuals undergoing this specialized training exhibited improved walking speed and smoother movements. The most significant enhancements were observed when participants engaged in this training 3 times a week over a month. In essence, proprioceptive training strategies with dual-task exercises proved effective in enhancing specific parameters related to walking, such as walking speed and other relevant aspects, among individuals with chronic stroke. This method aims to improve proprioceptive function, enhancing individuals’ ability to perceive and control their body’s positioning and movement, thereby addressing proprioceptive dysfunction and facilitating better functioning in daily life. By emphasizing the importance of proprioception in daily tasks, our study highlights the value of this approach to improving proprioceptive dysfunction after stroke.

Adapting one’s gait to environmental circumstances, such as avoiding obstacles and ensuring safe foot placement in cluttered environments, is essential for safe everyday walking (1). It is closely related to proprioception, defined as the sensations of one’s own body, including the sense of joint position, the perception of movement direction, velocity, distance, and timing, muscle force or tension, and effort (2, 3). Indeed, proprioceptive sensations provide feedback on the consequences of motor output from sensory receptors in the muscles, joints, and skin and are essential to the control of voluntary movement (3). The ability to transfer bodyweight from one leg to the other is a fundamental aspect of human locomotion and everyday activities. Moreover, the ability of persons with stroke to respond correctly to various environments and activities is impaired related to a decline in left/right weight transferability, proprioceptive dysfunction (4), and anticipatory responses (5). This transfer necessitates postural adjustments and is crucial for both gait and maintaining balance during activities of daily living (ADL). In this context, anticipatory postural adjustments involve the activation of postural muscles in a feedforward modality before the initiation of a voluntary movement, anticipating the destabilizing forces associated with the movement (6). An activity that qualifies as an anticipatory adjustment occurs when a subject is capable of conceptualizing and understanding the execution of a movement pattern for a specific situation. Regrettably, individuals who have experienced a stroke tend to place more weight on the non-affected leg and have a diminished ability to transfer weight within their base of support without risking a loss of balance (7). In particular, the simultaneous engagement in cognitive and motor performance – essential for most activities of daily living (ADL) – poses a significant challenge for these individuals (8).

The incidence of falls after discharge from rehabilitation programmes among stroke survivors is high (70%) (9). Over 12 months, 28% of individuals report falls at least once (10). At 5 years after a stroke, 88% of individuals could move independently indoors and outdoors, without assistance (11).

Moreover, gait speed decreases during dual**-**task exercises, particularly in the earlier stages following a stroke (12). Specifically, gait speed interference related to cognitive tasks is highly prevalent after stroke and often persists when the activity of simply walking improves (12). Considering these factors, speed can be regarded as a holistic parameter that integrates all gait parameters (13). Specifically, an adequate walking speed of 0.8 ms^−1^ or greater is necessary to enhance dual-task walking after stroke. Therefore, only individuals with sufficient walking capacity have the potential to improve dual-task walking (14). As a negative feedback loop, patients who fail to regain good speed may struggle with dual-task activities. This inability places them at a higher risk of falls and subsequent disability.

To improve balance, walking, and attention distribution during dual-task exercises, it's recommended to include proprioceptive training. This training aims to enhance the body's ability to sense its location, movement, and actions, thereby restoring its sensorimotor function (15). It centres on utilizing somatosensory signals, including proprioceptive or tactile afferents, in the absence of information from other modalities such as vision (15), to obtain the sense of positioning and movement. Proprioception training incorporates targeted exercises with the goal of enhancing an individual’s perception and control of their body’s position and movement in space. Crucial in neurological rehabilitation after stroke, proprioception training aims to enhance balance, gait, and overall sensorimotor function through a focused and varied set of exercises. A recent systematic review collected data on different strategies that combined proprioceptive training and dual-task exercises (16). It evaluates their effectiveness in improving balance and restoring gait in persons with stroke (16). Incorporating both proprioceptive training and dual-task exercises, the rehabilitation approach aimed at stimulating somatosensory signals through single- or multi-joint passive and active movement, somatosensory stimulation, and discrimination training (15). Proprioceptive training included: (a) maintaining balance on a treadmill, focused on stimulating proprioception to adjust walking pace, considering the tuning of the mobile platform (1, 17–22); (b) standing balance on an unstable pad (23) or overground walking, involving whole-task practice with different complexities, such as propulsion in various directions (4, 24–29), speed changes (30), wearable weights (31), resistance variations (32), body tilting in virtual reality (33), or aquatic games (34); and (c) proprioceptive neuromuscular facilitation techniques (35) (Table I). Simultaneous visual stimuli and proprioceptive feedback during gait training were considered effective after stroke (36). Cognitive and motor dual-task exercises, performed concurrently with proprioceptive training, significantly influence gait and balance, and enhance functional independence in stroke patients (18).

**Table I:** Stroke rehabilitation

Balance training	Proprioceptive exercises
Standard weight-bearing exercises (24; 48)	Maintaining balance on a treadmill, adjusting walking pace, or adapting to the tuning of the mobile platform (1; 17; 18; 19; 20; 21; 22)
Train balance (24; 49)	Standing balance on unstable pad (23)
Range of motion exercises (48)	Maintaining balance despite propulsion in various directions (4; 24; 25; 26; 27; 28; 29) , and resistance variations (32)
Isolated muscle strengthening exercises (24)	Balance during speed changes (30) and wearable weights (31)
Stretching exercises (24; 31; 49)	Balance during aquatic games (34) and body tilting in virtual reality (33)
Coordination exercises (24)	Proprioceptive neuromuscular facilitation techniques (35)
Gait training (18; 25; 31; 48; 49)	Simultaneous visual stimuli and proprioceptive feedback during gait training (36)

The dual-task exercises encompassed: (a) cognitive activities, using auditory (35) or visual cues (32), arithmetic operations (1, 17, 18, 21, 23, 27), counting backwards (1), word matching (24), verbal fluency exercises (26), memory tasks (20, 28, 33), exercise imagery (4), and planning activities (22); (b) motor activities, carrying objects (cups, coins, sandbag, or balls) (17, 19, 23, 25, 29, 31, 34), obstacle avoidance (1), playing Wii Fit games (33), and writing (30).

During the performance of dual tasks, observational and instrumented gait analyses serve as useful tools to assess walking abnormalities and fall risks. These analyses guide the development of specific interventions, help prevent further functional decline, and monitor rehabilitation outcomes (37). However, to date, there is still insufficient evidence on the significance of proprioceptive training.

Therefore, this systematic review aimed to analyse the effectiveness of specific proprioceptive training strategies with dual-task exercises.

## MATERIALS AND METHODS

### Study protocol

This systematic review was conducted in accordance with the Preferred Reporting Items for Systemic Reviews and Meta-analyses (PRISMA) statement (38), and following the Population, Intervention, Comparison, Outcome, and Study Design (PICOS) criteria (39) (see Table II for further details).

**Table II:** Summary of included studies

First author, publication year	Study design	Groups (*n*), age (years)	Months after stroke	Outcomes, gait parameters	Instrumented system	Proprioceptive training strategies with dual-task exercises	Therapy duration	Results after therapy
Ada 2003 (24)	RCT	A: 13 p (8 right/5 left hemiparesis) B: 14 p (6 right /8 left hemiparesis) 66±11 year	> 6	Speed, distance, step length, step width, cadence	Not specified walkway	A: cognitive task and walking programme (Matching the word “red” with the response “yes” or the word “blue” with the response “no” during an overground walking programme) B: low-intensity home exercises (exercises to lengthen and strengthen lower-limb muscles as well as to train balance and coordination)	A: 30 min, 3 d/w, for 4 w B: -, 3 d/w, for 4 w	The treadmill and overground walking programme was effective in improving walking. Gain of 18 cm/s in subjects with chronic stroke
Baek 2021 (18)	RCT	A: 16 p (10 right/6 left hemiparesis) B: 15 p (8 right/7 left hemiparesis) –	56.31±21	Speed, stride, variability, cadence	OptoGait (Microgate)	A: Gait training on a treadmill and cognitive task exercises at the same time (mental tracking, verbal fluency, executive function while standing) B: separately, before gait training on a treadmill, then cognitive task exercises	A and B 60 min, 2 d/w, for 6 w	DT gait treadmill training was more effective in improving gait ability in DT training and DT interference than single-task training involving gait and cognitive task separately in people with chronic stroke
Iqbal 2020 (31)	RCT	A: 32 p (14 right/18 left hemiparesis) 58.2±7.13 years B: 32 p (13 right/19 left hemiparesis) 58.8±6.13 years	Chronic stroke	Step length, stride length, 10 MWT, TUG, cycle time, cadence	Not specified walkway	A: motor DT exercises during proprioceptive training (slowly walking backwards, sideways, and forwards on a smooth surface while holding a 100g sandbag, and picking up plastic cups that lie in front of their feet while rising from a chair) B: conventional training (mat activities, stretching and strengthening exercises, and gait training)	A and B 40 min, 4 d/w, for 4 w	Conventional rehabilitation and DT training effectively improved gait ability of people with chronic stroke, and the latter showed significant improvement in all spatial and temporal gait variables compared with the former
Kim 2013 (25)	OS	A:14 p B: 15 p 56.4±12.3 years	7±2.4	Cadence, gait velocity, step time, cycle time, step length, and stride length	GAITRite system	A: single-task rehabilitation (rising from a chair, walking naturally on a flat surface forwards, backwards, and sideways, and going up a slope and stairs) B: DT motor training during proprioceptive training (aquatic proprioceptive training, adding the following exercises: getting up from a seated position while collecting plastic cups lying at their feet, then slowly walking forwards, sideways, and backwards on a flat surface while holding a 100 g sandbag against the affected wrist, and going up and down a ramp or stairs while transferring cups from tables of different heights located beside the ramp or stairs in consecutive order)	A and B 30 min, 5 d/w, for 4 w	Significative improvement in the temporal variables of cadence, gait velocity, step time, and cycle time, as well as in the spatial gait variables of step length and stride length in DT group
Liu 2017 (49)	RCT	A: 10 p (6 right/4 left hemiparesis) B: 9 p (4 right/5 left hemiparesis) C: 9 p (5 right/4 left hemiparesis) 50.2±10.7	40.8±33.3	Gait speed, cadence, stride time, stride length	GAITRite system	A: cognitive DT gait exercises during proprioceptive training (walking counting backwards by 3 digits) B: motor DT gait exercises during proprioceptive training (walking while carrying a tray with a bottle of water with the non-affected hand) C: conventional rehabilitation (strengthening, balance, and gait training)	A , B and C 30 min, 3 d/w, for 4 w	Cognitive training improved cognitive DT gait performance
Plummer 2021 (27)	RCT	A: 17 p (9 right/8 left hemiparesis) B: 19 p (6 right/13 left hemiparesis) –	< 3 years	gait speed	LEGSys™, Biosensics, System	A: DT gait exercises during proprioceptive training (training with obstacles) B: cognitive DT during single-task gait training Proprioceptive training strategies with cognitive dual-task exercises: dpontaneous speech, arithmetic, word generation, backward spelling, working memory, random number generation, calculating time, backward number recitation, naming opposites	A and B 30 min, 3 d/w, for 4 w	Both single- and DT-gait training improved single and DT gait speed but did not change the amount of relative interference
Saleh 2019 (34)	RCT	A: 25 p (13 right/12 left hemiparesis) B: 25 p (12 right/13 left hemiparesis) 49.7±1.8 years	9.02±1.8	Speed, step length, time of support on the affected limb	Biodex Balance	A: motor DT training in water during proprioceptive training (suspended chair and plinth, side bars at sides of the pool and balance board) B: motor DT training on land during proprioceptive training (standing on a balance pad while holding a cup containing water, walking at a comfortable speed with the slow movement of the non-paretic arm, while holding a ball, or a 200 mL cup of water in the non-paretic arm hand)	A and B 45 min, 3 d/w, for 6 w	Significant improvement in training in water rather than on land overall, anteroposterior and mediolateral stability index, walking speed, step length, time of support on the affected limb
Sengar 2019 (50)	CLT	A: 15 p (7 right/8 left hemiparesis) B: 15 p (6 right/9 left hemiparesis) 55.7±5.2 years	8.5±0.1	Speed, step length, stride length	Not specified walkway	A: DT during proprioceptive training with a fixed priority instructional set B: DT during proprioceptive training with variable priority instructional sets Proprioceptive training strategies with cognitive dual-task exercises: counting forwards and backwards by adding 3 to the digits Proprioceptive training strategies with motor dual-task exercises: walking with added difficulties, such as stepping forwards, backwards, and sideways.	A and B 45 min, 3 d/w, for 4 w	DT training with variable priority instructional sets was more effective than DT training with fixed priority instructional sets in improving gait parameters such as gait speed, stride length, and step length in people with chronic stroke
Seo 2012 (53)	OS	A: 20 p (12 right/8 left hemiparesis) B: 20 p (10 right/10 left hemiparesis) 63.57±5.9	6.9±2.4	Speed, cadence, step length, stride length, limb support period	Not specified walkway	A: motor DT exercises during proprioceptive training (standing on a balance pad and moving a cup containing water) B: single-task training (standing on a balance pad and moving a cup containing water)	A and B 30 min, 5 d/w, for 4 w	DT group showed a more significative change in sway area and maximum speed than the other group
Shim 2012 (48)	OS	A: 17 p (14 right/3 left hemiparesis) B: 16 p (10 right/6 left hemiparesis) 63.5±5.9	16.8±3.1	Speed, cadence, step length, stride length, limb support	GAITRite system	A: motor DT during proprioceptive training (kicking a ball, during proprioceptive training) B: traditional rehabilitation (range of motion exercises, functional mobility training, and gait training)	A and B 30 min, 3 A: 5d/w, for 6 w + 3 d/w for 6 w B: 5 d/w, for 6 w	DT improved gait ability
Yang 2007 (29)	RCT	A: 12 p (6 right/6 left hemiparesis) B: 13 p (10 right/3 left hemiparesis) 59.3±11.8	48.9±37.5	Speed, cadence, stride time, stride length, and temporal symmetry index	GAITRite system	A: DT motor exercises during proprioceptive training (walking at different speeds, in different directions (walkway, walking backwards) and with obstacles) B: no rehabilitation training	A: 30 min, 3 d/w for 4 w	Walking ability was significantly improved after training. Gain of 29.74 cm/s

p: patients after stroke; c: control group; A: group A; B: group B; y: years; f: female; m: male; d: days; h: hours; m: months; min: minutes; w: weeks; CLT: clinical trials; RCT: randomized controlled trials; OS: observational study; DT: dual-task; RT: retrospective study; TUG: Timed Up and Go test, 10MWT: 10 m walk test.

### Search strategy and data extraction

A systematic literature search was conducted between November 2020 and February 2022, with the last update in June 2023, across PubMed, Scopus, and Web of Science. The search was refined to include clinical trials in English, without any date restrictions.

The search terms included: “stroke” AND “rehabilitation” OR “training” OR “exercises” AND “proprioception” OR “proprioceptive” AND “dual-task” OR “task-performance” AND “gait analysis” OR “quantitative gait” OR “gait parameters”, used as both text words and keywords (Table III). The reference lists of relevant articles were also reviewed to include other suitable studies. Unpublished literature was excluded.

**Table III:** Table of terms and selection criteria

Study element	Components required for inclusion	Keywords	MESH terms
Stroke	Adult participation (≥ 18 years old); confirmed stroke diagnosis in chronic phase	“stroke”	“Stroke”
Rehabilitation	Proprioceptive training and dual-task exercises	“rehabilitation” OR “training” OR “exercises”	Rehabilitation
Proprioception	Stroke rehabilitation	“proprioception” OR “proprioceptive”	Proprioception
Dual-task	Gait parameters (speed, cadence, stride time, stride length, step length)	“dual-task” OR “task-performance”	Task performance
Gait parameters	Clinical trials and observational studies	“gait analysis” OR “quantitative gait” OR “gait parameters”	Gait analysis
Study details	Available in English		

To assess relevance based on predetermined inclusion criteria, two authors (RC and MC) independently performed data extraction, screened titles and abstracts, and collected information. In cases of disagreements, consensus was reached through discussion between the authors, with other authors consulted if necessary (for additional information on authors consulted or specific details for study selection and consulted sources, refer to Table IV).

**Table IV:** Protocol template of University of Warwick for systematic review

Background		
Important characteristics	Population and disease characteristics	Adult population with chronic stroke
Relevance	Implications for health	Clarification of the efficacy of specific proprioceptive training strategies with dual-task exercises for the recovery of proprioception, postural control, walking ability, and autonomy after stroke
Rationale	Evidence	Few studies in the literature on gait analysis and specific proprioceptive training
Justification of the search	Potential health implications	Interest to identify gait improvements related to proprioceptive training and to develop specific rehabilitation interventions for the improvement of postural stability and walking
Specification	PICOS components of the review question	Participants: older adults with chronic stroke; 59.22±6.46 y Intervention: rehabilitation therapy with proprioceptive training strategies with dual-task exercises Comparator: gait parameters Outcomes: clinical scales: TUG, BBS, 10MWT; gait parameters: gait speed, cadence, stride time, stride length, and step length Design: RCT, prospective and retrospective studies
Methods		
Search strategy	Electronic databases	PubMed, Scopus, and Web of Science
Key search terms		“stroke” AND “rehabilitation” OR “training” OR “exercises” AND “proprioception” OR “proprioceptive” AND “dual-task” OR “task-performance” AND “gait analysis” OR “quantitative gait” OR “gait parameters”
Other sources		Reference list of the considered articles to identify any other suitable documents
Selection criteria	Inclusion /exclusion criteria	Inclusion criteria: Design: clinical trials and observational studies Language: original article in English Participants: adults with a confirmed diagnosis of chronic stroke Intervention: rehabilitative programme with proprioceptive training strategies with dual-task exercises Comparison: no intervention, or other stroke rehabilitation Outcomes: changes in gait parameters Exclusion criteria: animal studies, papers with neurological diseases different from stroke
	Additional criteria of exclusion	Unpublished data, duplicates
Study selection		Two authors independently for the extraction of the studies and the data analysis
Quality assessment	Criteria for methodological quality	GRADE guideline
Presentation of extracted data		PRISMA, PICOS criteria
Data synthesis		Systematic review
Process		
Resources to conduct the review	Relevant expertise	Physicians
	Computing facilities	Microsoft Office
	Research databases	PubMed, Scopus, and Web of Science
	Bibliographic software	Citation software of Microsoft Word
Disseminated findings		Publication
Timetable		
Protocol for internal review	January 2020	MV, RC, MC
Protocol for external review	November 2020	MV, RC, MC
Searching and study selection	November 2020–September 2022	RC, MC, MV
Data extraction	December 2020–February 2022	EB, PF, SD
Quality assessment	January 2023	MV, RC, MC
Last update	June 2023	MV
Draft report for peer review	June 2023	dSA, MV, GLM
Submit for publication	June 2023	RC

TUG: Timed Up and Go; BBS: Berg Balance Scale; 10 MWT: 10 Meter Walk Test.

### Selection criteria and study selection

The data extraction, displayed in Table II, includes study design, sample size, participant characteristics, rehabilitative exercises, and gait parameters obtained by gait analysis.

Based on the eligibility criteria and PICOS guidelines, the included studies were 9 randomized clinical trials, 1 not randomized, and 2 observational studies; only original articles in English were included. The participants were all adults with a confirmed diagnosis of chronic stroke; the rehabilitative treatment comprised proprioceptive training strategies with dual-task exercises; the control groups (comparators) were either untreated patients or those given other exercises; the outcomes included gait parameters.

Exclusions were made for animal studies, papers on neurological diseases other than stroke, rehabilitation programmes different from proprioceptive training strategies with dual-task exercises, unpublished data, and stroke conditions different from chronic.

Inclusion criteria encompassed studies explicitly addressing dual-task exercises. The incorporation of dual-task elements was considered as it represented a specific category of comparison. Detailed distinctions between proprioceptive training, along with cognitive and motor dual-task exercises, and other stroke rehabilitation are presented in Tables I and II for clarity and comprehensive understanding.

**Fig. 1:**
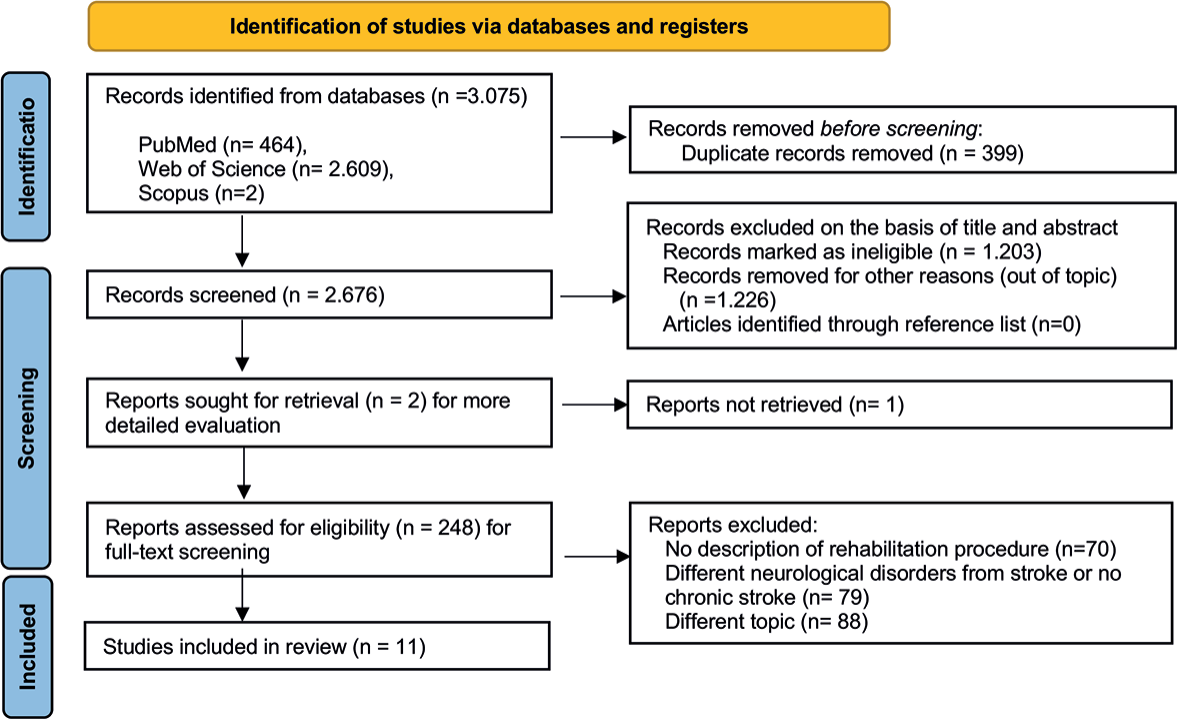
Flowchart of the process of initial literature search and extraction of studies meeting the inclusion criteria.

From the 3,075 records identified in the database, 248 papers were reviewed independently by the authors (RC, MC) after excluding duplicates, ineligible, and out-of-topic studies. Eleven publications met the inclusion criteria and were considered eligible for the present study. The remaining articles were excluded for the following reasons: 70 did not describe any rehabilitation procedure, 79 focused on neurological disorders other than stroke, and 88 did not examine gait parameters (Fig. 1).

For quality assurance, the research and data extraction processes were repeated by a third author (MV) during the last update in June 2023.

### Methodological quality and risk of bias

**Fig. 2:**
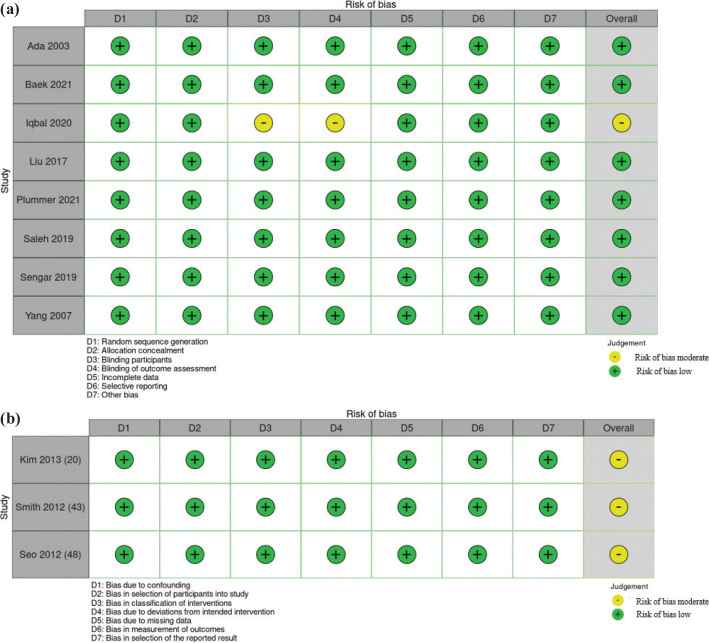
(a) Risk-of-bias summary: traffic-light plot for randomized controlled trials. (b) Risk-of-bias summary: traffic-light plot for non-randomized studies.

The evaluation of methodological quality was conducted by 2 independent reviewers (RC and MC), and any discrepancies were resolved through discussion or consultation with a third reviewer (MV). To ensure the reliability and validity of the included studies and to evaluate the level of evidence and quality, the Grades of Recommendation, Assessment, Development, and Evaluation (GRADE) approach (40–44) was used by 2 of the authors (RC and MC). The 2 authors independently used the Cochrane risk of bias tool for randomized controlled trials (45) and non-randomized studies (46). The quality and risk levels – classified as low, unclear, or high – were determined by analysing random sequence generation, allocation concealment, blinding of participants and personnel, outcome assessment, incomplete outcome data, selective reporting, and other potential biases (Table V, Fig. 2).

**Table V:** Review authors’ judgement regarding each risk of bias for each included study

First author, publication year	Random sequence generation	Allocation concealment	Blinding participants	Blinding of outcome assessment	Incomplete data	Selective reporting	Other bias
Ada 2003 (24)	+	+	+	+	+	+	+
Baek 2021 (18)	+	+	+	+	+	+	+
Iqbal 2020 (31)	+	+	–	–	+	+	+
Liu 2017 (49)	+	+	+	+	+	+	+
Plummer 2021 (27)	+	+	+	+	+	+	+
Saleh 2019 (34)	+	+	+	+	+	+	+
Sengar 2019 (50)	+	+	+	+	+	+	+
Yang 2007 (29)	+	+	+	+	+	+	+
First author, publication year	Bias due to confounding	Bias in selection of participants into study	Bias in classification of interventions	Bias due to deviations from intended intervention	Bias due to missing data	Bias in measurement of outcomes	Bias in selection of the reported result
Kim 2013 (25)	+	+	+	+	+	+	+
Smith 2012 (48)	+	+	+	+	+	+	+
Seo 2012 (53)	+	+	+	+	+	+	+

The heterogeneity of included studies was assessed considering study design, duration and timing of the rehabilitation, initial examination timings, assessments tools and specific gait parameters, and final outcomes (Table II).

## RESULTS

### Description of the studies and variations of experimental conditions across the studies

Fig. 1 illustrates the search strategy using the PRISMA chart. Table II shows the characteristics of the 11 studies included in the systematic review.

Across the 11 papers included in the systematic review, from a total of 393 stroke patients, 241 participants underwent proprioceptive training strategies with dual-task exercises, while 152 participants engaged in other stroke rehabilitation. Among the 241 participants who underwent proprioceptive training strategies with dual-task exercises, the distribution of tasks was diverse. Specifically, 71 participants engaged in cognitive dual tasks, while the majority, comprising 170 participants, participated in motor dual tasks. This nuanced breakdown highlights the variability in the types of dual-task activities undertaken within the studies included in the systematic review. Of the studies listed, all reported significant effects on gait after proprioceptive training strategies with dual-task exercises reported. The mean sample size across the 11 studies is approximately equal to 34.82. The group size varied between 19 and 64 participants.

The average duration of motor dual-task training across these studies is approximately 35 min per session, with an average frequency of 4 days per week, lasting for around 4.33 weeks (25, 29, 31, 34, 47, 48). The average duration of cognitive dual-task training across these studies is approximately 40 min per session, with an average frequency of 2.67 days per week, lasting for around 4.67 weeks (18, 24, 27). The average duration of motor and cognitive dual-task training across these studies is approximately 37.5 min per session, with an average frequency of 3 days per week, lasting for around 4 weeks (49, 50). Motor dual tasks included walking, slow movements, carrying objects, walking backwards, sideways, and forwards, picking up objects, walking at/in different speeds and directions, and walking with obstacles on the ground. Cognitive dual tasks include mental tracking, verbal fluency, executive function, word matching, counting backwards and forwards, arithmetic, word generation, backwards spelling, working memory, random number generation, calculating time, backwards number recitation, and naming opposites.

Table V and Fig. 2a and 2b present both the risk of bias assessment and the quality evaluation of the studies based on the GRADE guidelines. The population under study comprised adults affected by chronic stroke, > 6 months from the acute event (average months from acute event: 24.27 ± 15.53) (51, 52), and the intervention involved proprioceptive training strategies with dual-task exercises, with a comparison with other stroke rehabilitation (18, 24, 25, 27, 31, 49, 53, 54). Outcomes focused on the improvement of balance, gait, autonomy, and changes in gait parameters. The majority of studies (18, 24, 25, 27, 29, 34, 48–50, 53) exhibited a low risk across all evaluated domains. However, in 2001 Iqbal et al. (31) demonstrated a moderate risk, particularly in blinding of participants and outcome assessment. The quality assessment of the cumulative evidence across these studies revealed no significant limitations, inconsistency, or indirectness. Publication bias was deemed unlikely. The overall quality of evidence, assessed using the GRADE framework, was deemed high, indicating a high level of confidence in the systematic review’s findings.

For mapping out the research plan, a template from the University of Warwick was used, as indicated in Table IV.

Considerable heterogeneity was observed in the general clinical characteristics, including clinical presentation, severity of impaired postural control, and the specific gait parameters investigated (see Table II). A wide heterogeneity also existed among the studies regarding the disease duration, timing of the initial impaired postural control examination, and rehabilitation treatment duration.

The included studies primarily used instrumental gait analysis to assess the effectiveness of rehabilitation; only a few incorporated additional scales, such as Timed Up and Go (TUG) (18, 30, 31), Berg Balance Scale (BBS) (18), or 10 Meters Walking Test (10 MWT) (31) (Table II).

The most used was the GAITRite system (25, 29, 48, 49) and the OptoGait (Microgate) (18), utilized exclusively for gait analysis, and Biodex Balance System (34) and LEGSys™, Biosensics, System (27) used also for training.

Sengar et al. 2019 (50) , Seo et al. 2012 (53) Ada et al.2003 (24), and Iqbal et al. 2020 (31) did not specify the instrumental devices they used to assess gait parameters.

The heterogeneity related to the study design did not allow us to obtain enough quantitative results to conduct a meta-analysis, such as the different parameters related to gait measured across the small number of existing reports.

### Comparing studies: proprioceptive training strategies with dual-task exercises

The proprioceptive training, described in the included studies, was conducted in various ways: (1) on a balance pad (53); (2) on a treadmill, where proprioception was stimulated to maintain the walking pace based on the tuning of the mobile platform (18, 24); (3) on a flat surface (25, 27, 29, 31, 48–50), during overground walking (24), or while walking in water (34). The exercises required participants to adapt their gait to complete specific tasks such as moving forwards, backwards, sideways, walking up and down stairs, adapting to an irregular surface, or swaying (24, 25, 31, 34, 49, 50).

Table II outlines the proprioceptive training strategies with dual-task exercises. Most of the studies used a motor dual-task activity (25, 29, 31, 34, 48, 53) in conjunction with proprioceptive training, while a cognitive task was used in 3 studies (18, 24, 27). Two studies used both motor and cognitive tasks (49, 50).

Goal-oriented training was integrated into the dual-task exercises, such as walking while picking up a cup of liquid, for 4 weeks (25, 31, 49, 53), or while holding or kicking a ball, or a cup of water, for 6 weeks (34, 48). Cognitive tasks included mental tracking, verbal fluency, and mathematical or executive functioning while standing, conducted for 4 weeks (27, 49, 50) and 6 weeks (18). For instance, exercises involved walking while avoiding obstacles (29), or identifying colours (24).

### Comparing studies: the stroke rehabilitation of the control group

Dual-task exercises during proprioceptive training are part of the training regimen for stroke patients. To better understand their role and potential, several articles included in the review compared them with other exercises. The control groups engaged in various rehabilitation interventions: low-intensity home exercises (24), muscle strengthening exercises (24), coordination exercises (24), stretching (24, 31, 49), standard weight-bearing exercises (24, 54), train balance (24, 49), range of motion exercises (54), and gait training (18, 25, 31, 49, 54). Single-task training focusing on balance control and motor function without dual-task demands (18, 27, 53).

### Implications for rehabilitation

The primary goals of rehabilitation include reducing the risk of falls, maintaining functionality in activities of daily living (ADL), preserving postural control, and mitigating the severity of stroke-related symptoms (55). Dual-task exercises that require cognitive engagement can enhance motor learning for posture and gait control, thereby improving balance, motor control ability, and proprioception (56).

Neurological rehabilitation in stroke patients is of utmost importance. Thus, stroke rehabilitation programmes are adapted to patients’ clinical conditions and comorbidities, which might include conditions such as neurologic bladder (47, 57), or dysarthria (58, 59), and not only motor disorders. This approach helps to minimize interruptions, with a holistic perspective of stroke patients. Additionally, while the benefits of specific nutraceuticals are still under study, they could potentially improve the adherence to rehabilitation (60, 61) and are often added as an integral part of recovery programmes. Thus, while stroke rehabilitation already addresses various stroke-related impairments, the uniqueness of the proposed programme lies in its intentional combination of proprioceptive training strategies with dual-task exercises with a goal-oriented final aim (carrying an object while walking or telling a story while keeping balance). This integration plans to target both sensorimotor and cognitive aspects concurrently, potentially offering enhanced benefits for individuals recovering from stroke, reducing the risk of falls and postural imbalance during ADL.

Future research is expected to refine proprioceptive training programmes, developing new exercise sets, incorporating smart technologies for self-guided rehabilitation, and devising new methods for more specific diagnoses of proprioceptive impairments. Additionally, it is vital to ensure patient safety throughout the treatment, by creating safe rehabilitation environments.

## DISCUSSION

### Summary of collected data

This systematic review reports data related to improvements in gait parameters following proprioceptive training strategies with dual-task exercises in people with chronic stroke. Indeed, despite the different approaches to rehabilitation, the current literature concurs that proprioceptive training strategies with dual-task exercises effectively aid in the improvement of important gait parameters in chronic stroke patients. To our knowledge, no other published studies have reviewed these data on gait parameters after proprioceptive training strategies with dual-task exercises in a chronic stroke population. All the studies analysed involved clinically stable patients in the chronic stage of stroke, who underwent proprioceptive training strategies with dual-task exercises.

Regrettably, the current literature is limited, offering no definitive evidence to suggest which proprioceptive training strategies with dual-task exercises are superior, when it should be initiated, or their optimal duration and intensity. Despite the limited number of studies included in this systematic review, the results underscore important clinical implications. They highlight the potential value of incorporating dual-task exercises in proprioceptive training to reduce the risk of falls and improve gait and autonomy in people with chronic stroke, even when performing 2 tasks simultaneously.

### Comparing studies: gait parameters

Hemiparetic gait is characterized by specific spatiotemporal patterns, including decreased cadence, prolonged swing duration on the paretic side, extended stance duration on the nonparetic side, and step length asymmetry, as compared with the gait parameters of healthy subjects (62). Gait speed is one of the most widely studied parameters, as indicated by numerous papers included in the systematic review (18, 24, 25, 27, 29, 34, 48–50). Significant changes in gait parameters, especially gait speed, have been documented in individuals with Parkinson’s disease (63), dementia (13), and multiple sclerosis (64). Perry et al. (65) provided compelling evidence that walking speed is predictive of community walking ability. The normal range of gait speed falls between 1.2 and 1.4 m/s. However, this can vary depending on age, gender, and anthropometrics. In older people, a walking speed of less than 0.8 m/s is often associated with limited mobility in community settings (66). In 1995, Perry et al. (67) demonstrated that a speed of less than 0.4 m/s predicts household walking; 0.4–0.8 m/s predicts limited community walking; and more than 0.8 m/s predicts unlimited community walking in stroke individuals (65). Furthermore, transitioning from one speed-based category to another is associated with improvements in self-reported measures of function and quality of life (68). Therefore, walking speed has been used to stratify patients with neurologic injury (69).

Moreover, comfortable gait speed is also linked to regular executive functioning. A higher walking speed is associated with better cognitive ability than a slower speed, reflecting optimal function of the locomotor system in patients affected by dementia (13). Conversely, poor physical function and slow walking speed are correlated with deteriorating cognitive function in older people (66). Individuals with chronic stroke often prioritize task accuracy and completion over maintaining walking speed. This behaviour is more pronounced during cognitive tasks than motor tasks, especially at maximum walking speed in stroke patients (70).

Thus, speed is a fundamental and clinically relevant gait parameter, commonly used to assess functional mobility and overall gait performance. Therefore, studies emphasized speed as a key outcome measure. Moreover, previous research trends and established practices in gait analysis could have influenced the choice of parameters studied, with speed often being a well-established measure. However, other gait parameters were investigated, but they varied across studies. Overall, the heterogeneity in parameter selection made drawing conclusive findings challenging.

The variability of gait pattern indices is considered a reflection of gait instability, and a risk factor for falling (71). The cause of this variability can be related to central nervous system impairments that can affect stance time variability, especially in slow walkers, while sensory impairments affect step width variability in fast walkers (72). Moreover, persons with stroke with very low gait speed (< 1.4 km/h or < 2.4 km/h) demonstrated longer stride, step lengths and lower cadence, potentially linked to a higher reliance on handrail use (62). Step length asymmetry also seems to be influenced by propulsive force-generation ability during hemiparetic walking (73). Thus, one of the mechanisms for the longer paretic step may be the relatively greater compensatory nonparetic leg propulsion, and asymmetrical step lengths may not necessarily limit the self-selected walking speed (73). This asymmetry, especially in high-gait speed individuals, seems to be more closely related to patients’ balance ability than to hemiparesis (62). Thus, gait training, improving gait parameters, can also positively influence balance.

Another aspect to be considered is that spatiotemporal gait asymmetry was more closely related to balance measures involving dynamic tasks than static tasks, suggesting that gait asymmetry may be related to the high number of falls after stroke (74). Thus, proprioceptive training strategies with dual-task exercises have the potential to reduce the frequent number of falls among stroke survivors.

As indicated in Table II, the most frequently analysed parameters included gait speed, cadence, and stride time (temporal parameters), as well as stride length and step length (spatial parameters). The frequent study of these spatio-temporal parameters led to more accurate results, while less reported parameters, such as stride time, increased the difficulty of establishing consistent findings.

After undergoing proprioceptive training strategies with dual-task exercises, people with chronic stroke tended to show positive changes in gait parameters, including increased gait speed (18, 24, 25, 27, 29, 34, 48–50), cadence (18, 24, 25, 29, 31, 48, 49), stride length (18, 25, 29, 31, 48–50), and step length (24, 25, 31, 34, 48, 50), along with a decreased stride time (29, 49). Single limb support time on the affected side (29, 34) and for both paretic and non-paretic leg (48) also improved after training (29, 34, 48). These improvements reflect the potential benefits of the training in enhancing the overall walking abilities of people with chronic stroke. Cadence (18, 24, 25, 31, 48, 49) and stride length (18, 25, 31, 48, 49) showed positive changes even with single-task proprioceptive training alone. Furthermore, a regular training regimen of 3 days a week for 4 weeks positively affected speed (24, 27, 29, 49, 50), cadence (24, 29, 49), stride length (29, 49, 50), and step length (24, 50). However, there was high heterogeneity among the studies in time variables, including the frequency and duration of training sessions.

The present results may contribute to showing the need for targeted rehabilitation to address compensatory strategies, encompassing both feedback and feedforward mechanisms, for the recovery of gait stability and reduction in a related risk of falls.

### Instrumented gait parameters vs clinical assessments

In many clinical settings, access to advanced treadmill equipment for gait training and gait analysis may be limited. Therefore, observational gait analysis remains a valuable tool for assessing patients’ gait patterns and functional abilities, as well as feasibility and efficacy, which are conventional, ground-based, and self-paced gait training methods.

While instrumented gait analysis offers detailed quantitative data, and precise measurements of spatiotemporal parameters, kinematics, and kinetics, observational analysis provides clinicians with important qualitative insights into gait abnormalities, functional limitations, compensatory strategies, and gait patterns used in real-world conditions (75).

While instrumented gait training offers precise control over gait parameters and feedback mechanisms, conventional gait training methods, such as overground walking or circuit-based exercises, remain widely used due to their accessibility and applicability in various clinical settings (76). Ground-based and self-paced gait training methods allow clinicians to address functional mobility and gait deficits in real-world contexts, simulating daily activities and environmental challenges more closely. Additionally, they often require minimal equipment and can be easily adapted to patients’ needs and abilities, making them practical and cost-effective alternatives to instrumented gait training to preserve walking pattern (76).

Baek et al. (18) and Kim et al. (25) showed the positive impact of proprioceptive training strategies with dual-task exercises in improving motor performance, balance, and gait, assessed with functional scales, in particular TUG (18, 30, 31), BBS (18), 10 MWT (31). Moreover, proprioceptive training strategies with dual-task exercises haves demonstrated significant efficacy in improving the walking abilities of people with chronic stroke, as evident in both instrumental gait parameters and clinical assessments. Instrumental measures highlight the positive impact of this training on objective gait metrics. Moreover, clinical assessments conducted through various functional scales consistently show enhanced motor performance, balance, and functional independence. This dual-pronged approach, addressing both instrumental and clinical aspects, underscores the comprehensive benefits of proprioceptive training strategies with dual-task exercises in the rehabilitation of people with chronic stroke.

### Study limitations

Concerning the limitations of the study, the lack of uniformity in study designs – particularly in terms of measured parameters related to gait and electronic instruments, as well as the dose of given intervention – and the small number of existing reports prevented a quantitative analysis. Moreover, included studies seldom reported clinical assessments. Furthermore, missing information on certain clinical characteristics, which could influence gait parameters, posed a confounding factor. Such characteristics include comorbidities affecting gait such as osteoarthritis, arthrosis, or peripheral neuropathies, use of medications or orthosis, and concurrent stroke-related depression. Many studies also failed to assess the educational status of the participants, which could potentially influence adherence to treatment and, thus, impact the results. Moreover, the variation in dual-task exercises, encompassing cognitive, motor, and combined cognitive-motor interventions, is acknowledged as a noteworthy limitation in systematic reviews. The inclusion of diverse dual-task approaches across the studies introduces heterogeneity in the interventions, making it challenging to isolate the specific effects of each strategy.

Moreover, the studies included in the review utilized various methods for gait analysis, including instrumental devices such as treadmills, as well as clinical functional scales like TUG, BBS, and 10 MWT to assess gait speed and stride length. It is important to acknowledge that the availability of advanced instrumental gait analysis tools may vary among clinicians, and many rely on more subjective observational gait analysis methods due to limited access to such technology. This limitation can have implications for rehabilitation practices, as objective measures of gait parameters are crucial for informing treatment decisions and monitoring progress effectively. Another limitation within the clinical context pertains to whether the intervention of “proprioceptive training with dual-task exercises” relies on specialized treadmill equipment that may not be readily available to many clinicians. This limitation poses significant challenges to the implementation and widespread adoption of such interventions in rehabilitation settings. Finally, the studies relied on instrumented gait analysis, and only a few on scales. Furthermore, the relatively limited sample sizes may be of concern when evaluating the effect of the rehabilitation approach of the included studies. Lastly, another limitation of the present review is that the included studies primarily compared 2 different groups, but follow-up assessments were not clearly reported.

### Conclusion

Balance control and walking ability during ADL are crucial rehabilitation goals and significant concerns for patients, families, and therapists alike.

Taken together, the findings of this systematic review suggest that proprioceptive training strategies with dual-task exercises might lead to improvements in temporal gait parameters such as speed, cadence, and stride time, as well as spatial parameters such as stride and step length, in chronic stroke populations during ADL.

Even if there is a limited number of studies in the current literature exploring gait analysis and specific proprioceptive training strategies with dual-task exercises, this systematic review serves a critical role in identifying potential gait improvements associated with proprioceptive training strategies with dual-task exercises. Additionally, it outlines specific rehabilitation interventions aimed at enhancing dynamic postural stability and walking during ADL, thereby reducing the risk of falls in the chronic stroke population.

Even though intervention using proprioceptive training strategies with dual-task exercises are promising for improving gait parameters in individuals with stroke, randomized controlled trials with larger sample sizes, standardized method, and outcome measures are required to evaluate the effectiveness of such a rehabilitative strategy.
